# Moderately Escalated Hypofractionated (Chemo) Radiotherapy Delivered with Helical Intensity-Modulated Technique in Stage III Unresectable Non-Small Cell Lung Cancer

**DOI:** 10.3389/fonc.2013.00286

**Published:** 2013-11-18

**Authors:** Vittorio Donato, Stefano Arcangeli, Alessia Monaco, Cristina Caruso, Michele Cianciulli, Genoveva Boboc, Cinzia Chiostrini, Roberta Rauco, Maria Cristina Pressello

**Affiliations:** ^1^Department of Radiotherapy, Azienda Ospedaliera S.Camillo-Forlanini, Rome, Italy; ^2^Department of Medical Physics, Azienda Ospedaliera S. Camillo-Forlanini, Rome, Italy

**Keywords:** dose escalation, hypofractionated radiotherapy, chemo-radiation, unresectable NSCLC, helical tomotherapy

## Abstract

**Purpose:** To assess clinical outcomes and toxicities in patients with stage III unresectable non-small cell lung cancer (NSCLC) treated with a moderately escalated hypofractionated radiotherapy delivered with Helical Intensity-Modulated Technique in combination with sequential or concurrent chemotherapy.

**Materials and Methods:** Sixty-one consecutive patients considered non-progressive after two cycles of induction chemotherapy were treated with a moderately escalated hypofractionated radiation course of 30 daily fractions of 2.25–2.28 Gy each administered in 6 weeks up to a total dose of 67.5–68.4 Gy (range, 64.5–71.3 Gy). Thirty-two received sequential RT after two more cycles (total = 4 cycles) of chemotherapy, while 29 were treated with concurrent chemo-radiation. The target was considered the gross tumor volume and the clinically proven nodal regions, without elective nodal irradiation.

**Results:** With a median follow up of 27 months (range 6–40), 1-year and 2-year OS rate for all patients was 77 and 53%, respectively, with a median survival duration of 18.6 months in the sequential group and 24.1 months in the concomitant group. No Grade ≥4 acute and late toxicity was reported. Acute Grade 3 treatment-related pneumonitis was detected in 10% of patients. Two patients, both receiving the concurrent schedule, developed a Grade 3 acute esophagitis. The overall incidence of late Grade 3 lung toxicity was 5%. No patients experienced a Grade 3 late esophageal toxicity.

**Conclusion:** A moderately hypofractionated radiation course delivered with a Helical Intensity-Modulated Technique is a feasible treatment option for patients with unresectable locally advanced NSCLC receiving chemotherapy (sequentially or concurrently). Hypofractionated radiotherapy with a dedicated technique allows safely dose escalation, minimizing the effect of tumor repopulation that may occur with prolonged treatment time.

## Introduction

More than two third of the patients with non-small-cell lung cancer (NSCLC) in the Western Countries are found to have locally advanced or metastatic disease at the time of diagnosis (1). Improving outcomes for patients with stage III disease still remains a major challenge. Concurrent chemo-radiation is the current mainstay of treatment for unresectable NSCLC, since two meta-analyses have confirmed the benefit of concomitant approach using platinum-based therapy (2, 3). Nevertheless, local control is achieved in 40% and 5-year survival is 15%. Other than systemic failures, these poor clinical results can be partly attributed to the still high rates of thoracic failures with traditional radiation doses and techniques that cannot allow to deliver the radiation doses beyond a certain threshold in order to avoid the risk of unacceptable toxicities. Indeed, while huge research have been devoted on improving systemic therapy options for patients with advanced lung cancer, less efforts have been placed on the importance of increasing the delivered radiation dose beyond 60 Gy, which has been the standard for over 20 years (4). Martel et al. (5) at the end of 1990s estimated that the dose to achieve a 50% local control at 2 years should be above 70 Gy. Soon after, improvements in radiation delivery techniques that have the potential to better sparing of normal tissues as well as advances in tumor volume definition have focused the attention in the investigation of dose escalation. By using a conventional fractionation regimen, however, dose escalation is obtained by increasing the number of daily treatments, thus resulting in a prolongation of the overall time. Unfortunately in NSCLC such a long duration of the radiation course has been shown to be detrimental to tumor control and survival, resulting in a significantly shortened survival (*p* = 0.016) in four Radiation Therapy Oncology Group (RTOG) prospective randomized trials (6), with a loss of survival rate of 1.6% per day of prolongation beyond 6 weeks (7). Therefore, both total radiation dose and treatment duration (or overall time) should be considered crucial factors affecting the outcome of radiotherapy in the management of NSCLC. Relying on a better conformal avoidance of normal healthy tissues obtained with image-guided rotational IMRT (8), we applied an alternative strategy that has already been shown (9) to effectively escalate the dose by increasing dose per day while reducing the number of treatment fractions and duration of the treatment course, thus avoiding the risk of lessening the benefit of the extra dose due to tumor cell repopulation during treatment (6, 10). In this article we retrospectively analyzed data from 61 consecutive patients with stage III unresectable NSCLC treated with a moderately escalated hypofractionated radiotherapy delivered with Helical Intensity-Modulated Technique in combination with sequential or concurrent chemotherapy.

## Materials and Methods

### Population

The analysis included 61 patients with stage III unresectable NSCLC who were considered non-progressive after two cycles of induction platinum-based chemotherapy, basing on a contrast-enhanced computed tomography (CT) scan of the chest, brain, and upper abdomen. The treatment policy was reviewed and approved by the IRB and carried out in compliance with the Helsinki Declaration of 1975, as revised in 2000. Written informed consent was obtained from each patient.

### Pretreatment evaluation

Initial workup included bronchoscopy, CT of the lung and upper abdomen through the adrenal glands, an MRI of the brain with contrast, and a bone scan. A whole body 18F-deoxyglucose (FDG) – Positron Emission Tomography (PET) scan was performed in 33 patients (54%). All patients had a Forced Expiratory Volume in the first second (FEV1) and DLCO (Carbon Monoxide Diffuse Capacity) at least 40% of predicted value, adequate blood tests, consisting in absolute neutrophil count >1500/mL, hemoglobin count ≥10 g/dL, platelet count ≥100,000/mL, serum creatinine level <1.6 mg/dL, serum bilirubin <1.5 times normal institutional limits, serum aspartate aminotransferase, and alanine aminotransferase <2.5 normal institutional limits, and a World Health Organization Performance Status (WHO-PS) ≤2. None of them experienced a weight loss of more than 10% in the last 6 months.

### Treatment

#### Treatment plan – chemotherapy

Induction chemotherapy consisted or cisplatin (80 mg/m^2^) or carboplatin (AUC 5) on day 1 and gemcitabine 1250 mg/m^2^ on day 1 and 8, for a total of two cycles repeated every 21 days. Thereafter, patients candidates for chemo-radiotherapy with curative intent were those considered non-progressive (RECIST criteria) (11). In a first phase, the treatment schedule consisted in a sequential approach, with radiation course intended to start at the end of two more cycles of chemotherapy following the same rules described above, within 7 days from day 21 of the cycle 4. After a first report (12) showing only minor complications, all non-progressive patients after induction chemotherapy were treated with concurrent chemoradiation. In the concurrent schedule cisplatin-vinorelbine (cisplatin 40 mg/m^2^ day 2 and 9, vinorelbine 15 mg/m^2^ day 2 and 9, cisplatin 40 mg/m^2^ day 23, vinorelbine 15 mg/m^2^ day 23 and 30) was used, and radiotherapy began within 7 days after the completion of induction chemotherapy (within 7 days from day 21 of the cycle 2).

#### Treatment plan – radiotherapy

##### Simulation

All patients were positioned supine on a wing board and immobilized by means of thermoplastic frames. CT scan for planning from the level of the cricoid cartilage through the whole liver volume was acquired in shallow breathing mode at 3 mm slice thickness, ensuring that the amplitude of respiration, that was checked under fluoroscopy, was kept within maximum 15 mm. The gross tumor volume (GTV) included the primary tumor and the pretreatment involved lymph nodes as defined on CT imaging (short axis >1 cm or necrosis) or on FDG-PET. For the clinical target volume (CTV) a margin of 5 mm incorporating microscopic disease around GTV was used (13). Depending on the tumor location, the planning target volume (PTV) included the CTV plus a total margin of at least 1 cm to the superior-inferior dimensions and at least 0.8 cm in the axial plane, unless the PTV expansion extended outside of the skin, or into the spinal canal. In this case, PTV margins were limited. Automatic contouring of the lungs and heart was performed using the Pinnacle3 treatment planning system (version 8.0 h; Philips Radiation Oncology Systems, Fitchburg, MA, USA), with manual corrections allowed. Planning risk volumes (PRV) were constructed with a 3-mm margin for the spinal cord and 5-mm for the esophagus.

##### Dose schedule, constraints, and treatment delivery

Dose prescription to the median dose point of the entire PTV was 30 fractions of 2.25–2.28 Gy each up to a total dose of 67.5–68.4 Gy (range, 64.5–71.3 Gy). According to the linear-quadratic model, the corresponding normalized total dose at 2 Gy per fraction (EQD2) is approximately 70 and 72 Gy, considering an alfa/beta ratio of 10 Gy for tumor and acutely responding normal tissues and 3 Gy for late complications, respectively. The optimization was driven with the aim of delivering the prescribed dose to at least 95% of the PTV, according to ICRU 50/62 guidelines (14). DVH’s points and penalties were setted to best meet the constraints for organs at risk (OARs) without compromising PTV coverage. Specific dosimetric guidelines for OARs in accordance to the Quantec (15) dose-volume model were applied and rescaled on fractionation’s change as follows: V19 for lungs <30%, MLD (volume of both lungs minus GTV) <19 Gy; a maximal dose (*D*_max_) of 47 Gy on the spinal cord; mean esophageal volume <32 Gy, V33 <50%, V47 <40%; mean heart volume <33 Gy, V38 <80%, V57 <30%. Dose computation and treatment delivery were performed on the TomoTherapy HiArt II system (TomoTherapy Inc., Madison, WI, USA). Image-Guided Radiotherapy (IGRT) was performed by means of a Megavolt Computed Tomography (MVCT) before each daily session in the same shallow breathing modality adopted on CT simulation, and positioning was done using the integrated registration with the planning CT to account for set-up uncertainties. The delivery parameters usually used for treatment planning and optimization were: 2.5 cm (field width); 0.287 (pitch); 2.5 (modulation factors); 0.215 cm × 0.215 cm; (dose calculation grid). Treatment replanning was never performed considering that tumor shrinkage during the radiation course is small and might be counteracted by the risk of delivering inadequate dose to the tumor rind, where residual cancer clonogens may still be present (16).

### Response and toxicity evaluation

Patients were seen weekly during treatment and at a 3-monthly interval during the first 2 years of follow up and every 6 months thereafter. Toxicity monitoring was focused on treatment-related esophageal and pulmonary adverse events and assessed by the RTOG grading system (17). Any increase in grade form baseline was considered toxicity related to the treatment and calculated for the acute (90 days from start of RT) and late phase (beyond 90 days). Assessment of tumor response relied upon RECIST criteria (11). Progressive disease that developed within or at the margin of the PTV, as well as recurrences in another lobe of the ipsilateral lung, was scored as loco-regional failure, whereas progression in the contralateral lung or extrathoracic sites was defined as distant failure. Overall survival was calculated by the Kaplan–Meier method from the initiation of treatment and patients were censored at the time of the specific event.

## Results

This report includes 61 enrolled patients with locally advanced stage III unresectable NSCLC treated between 2008 and 2011, with a median follow up of 27 months (range 6–40). All patients were considered non-progressive after two cycles of induction platinum-based chemotherapy. Among them, 32 received sequential RT after two more cycles (total = 4 cycles) of chemotherapy, while 29 were treated with concurrent chemoradiation. All patients but one, who discontinued treatment due to a decline in performance status, finished the scheduled course, with a median of 42 days (range, 42–45 days). One patient died prematurely from non-cancer and non-treatment-related causes within 3 months after completion of the radiation course. Details on the baseline disease, patients, and treatment characteristics are summarized in Table 1.

**Table 1:** **Baseline characteristics**.

	Value
**PATIENTS CHARACTERISTICS**	
*N* Patients	61
Age (year)	67 (Range 40–78)
Sex
Mal	46 (75.4%)
Female	15 (24.6%)
COPD
Yes	39 (64%)
No	22 (36%)
WHO-PS
0	22 (36%)
1	31 (50.8%)
2	8 (13.2%)
Smokers
Never	7 (11.4%)
Quit	46 (75.4%)
Current	8 (13.2%)
**DISEASE CHARACTERISTICS**	
Type of carcinoma
Adenocarcinoma	31 (50.8%)
Squamous cell	23 (37.8%)
Unspecified NSCLC	7 (11.4%)
Stage (TNM sixth edition)	IIIA 35 (57.4%)
	IIIB 26 (42.6%)
Median GTV size (cc)	81.8 (5.9–598.8)
Tumor location
Upper-middle lobes	47 (77%)
Inferior lobes	14 (23%)
**TREATMENT CHARACTERISTICS**	
Chemotherapy timing
Induction	All (100%)
Sequential	32 (52.5%)
Concurrent	29 (47.5%)
Drugs in sequential schedule
Cisplatin-gemcitabine	28 (87.5%)
Carboplatin based	4 (12.5%)
Drugs in concurrent schedule
Cisplatin-vinorelbine	29 (100%)
Total radiation dose	67.95 Gy (64.5–71.3 Gy)
Median OTT (days)	42 (42–45)

### Toxicity

No Grade ≥4 acute and late toxicity was reported. Acute Grade 3 treatment-related pneumonitis was detected in 10%. In all cases, acute lung toxicity developed 2–4 months after the completion of treatment and resolved within 7 months. Two patients, both receiving the concurrent schedule, developed a Grade 3 acute esophagitis. The overall incidence of late Grade 3 lung toxicity was 5%. No patients experienced a Grade 3 late esophageal toxicity.

### Local control and survival

**Figure 1:**
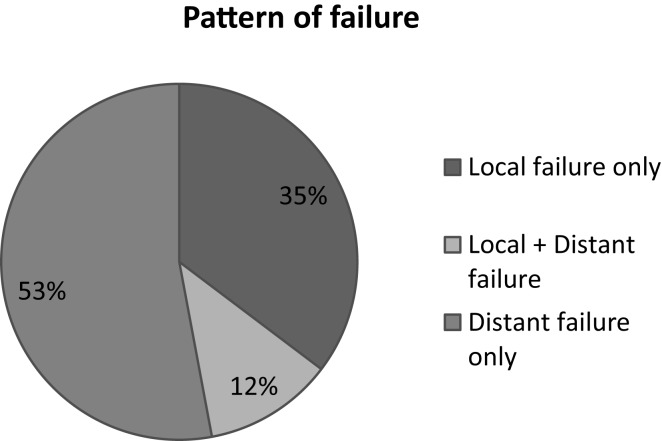
**Pattern of failure**.

**Figure 2:**
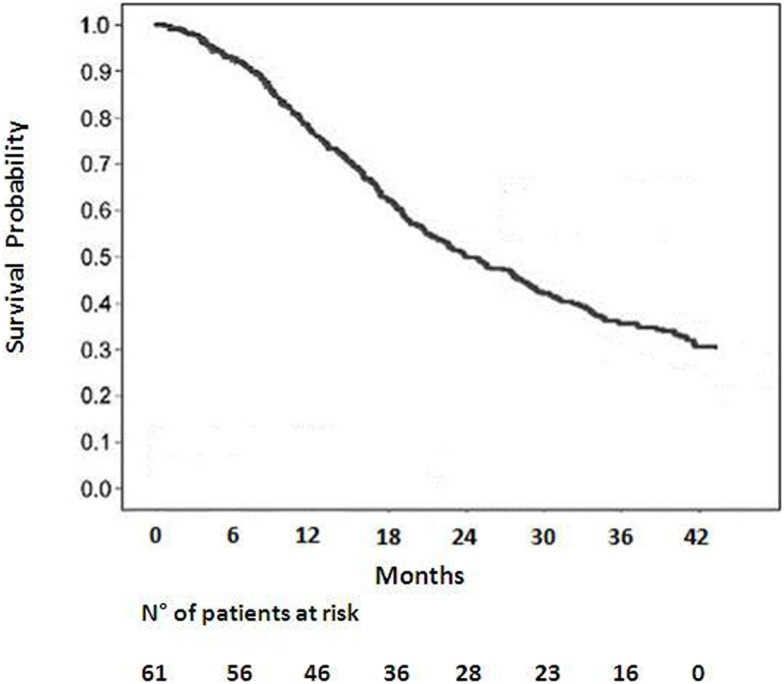
**Overall survival for all patients**.

Among 59 patients evaluable for local control, the overall response rate was 54% (6% CR, 48% PR). Stable disease was observed in 20%. Progression was documented in the remaining patients. The median survival duration was 18.6 months in the sequential group and 24.1 months in the concomitant group. A summary of the analysis of patterns of failure is provided in Figure 1. One-year and 2-year OS rate was 77 and 53% respectively for all patients (Figure 2), 43% of whom were stage IIIB.

## Discussion

The renewed interest in the adoption of dose escalated regimens has recently prompted the RTOG to open a randomized Phase III trial, RTOG 0617 (18), to determine whether chemo-radiotherapy with a higher radiation dose (74 Gy) improved overall survival compared with the current standard dose (60 Gy). Unexpectedly, early findings (19), demonstrated that the higher dose of radiation did not improve overall survival, and the study was closed to further participant enrollment in the high-dose arm. In absence of a difference between the toxicity rates between the two groups, it can be speculatively argued that at least two factors may be advocated for this disappointing outcome: (1) a higher risk of death related to the effects on the normal lungs and perhaps the heart from high-dose three dimensional conformal radiotherapy (3D-CRT) and IMRT; (2) the protraction of the overall treatment time beyond 6 weeks in the high-dose arm, that might have favored tumor repopulation. These poor results warrant the radioncological community to move a step backwards in the dose escalated approach. However the path for dose escalation should not be abandoned since local failure following concurrent chemotherapy and normo-fractionated radiation therapy for patients with stage III NSCLC approximates 85% (20), and the effect of higher radiation doses on survival is shown to be independent of whether chemotherapy is given (21). Thus, RT dose intensity remains important despite the establishment of chemotherapy in Stage III NSCLC, ensuring a 4% relative improvement in survival and 3% relative improvement *in loco*-regional control for every 1 Gy BED increase (22). Over last decade, radiotherapy schedules other than conventional fractionation have been explored for dose intensification in unresectable NSCLC: hyperfractionation has been investigated with promising results and its efficacy has been substantiated in a Meta-Analysis of Radiotherapy in Lung Cancer (MAR-LC) (23) conducted on 2000 patients affected with NSCLC that found that modified fractionation (accelerated or hyperfractionated radiotherapy) improved overall survival as compared to conventional radiotherapy, resulting in an absolute benefit of 2.5% (8.3–10.8%) at 5 years. Although increasing the RT dose intensity by accelerating the time may represent a suitable strategy, its application in the clinical practice may be challenging and limited by the logistic difficulties of treating patients multiple times in a day and an expected rates of greater acute esophageal toxicity. On the other hand, the administration of higher daily doses (hypofractionated RT) would be certainly be more attractive, allowing to complete the treatment in fewer fractions, but has long been discouraged given some concerns on the potentially increased late adverse effects. Mehta et al. (9) have developed a dose per fraction escalation schedule in NSCLC using advanced radiotherapy delivery technologies. The strength of this approach is the capability of escalating the dose by moderately increasing the dose per fraction without prolonging the duration of the treatment course beyond 6 weeks – which might counteract the benefit of dose escalation allowing time for the tumor to begin re-growing. We implemented this alternative strategy in the context of combined chemo-radiotherapy and we reported 10% Grade 3 acute lung toxicity, which is consistent with the 8 and 11% encountered by others (24–26) and even lower than major treatment-related pneumonitis rates observed in some recent trials that have assessed hypofractionated RT regimens in association with chemotherapy (27, 28). Our findings confirm that moderate hypofractionation using IGRT techniques, that help to reduce the total irradiated volume, might not actually increase the risk of radiation pneumonitis in typical “parallel” organs such as the healthy lungs – with an expected marked volume effect – despite the tumor fractionation sensitivity is smaller than that of the critical normal tissue (29). The time course of acute lung toxicity reflected the typical pattern of behavior of the classical radiation pneumonitis, having developed 2–4 months after the completion of radiation and resolved without sequelae within 7 months (30). Then, in two patients who experienced a Grade 3 acute esophagitis, the maximum time of discontinuation of treatment did not exceed 3 days, thus resulting in a very short treatment break. Late toxicity was mild, with no patients experiencing a >G2 esophagitis. Outcomes in terms of local tumor control and survival seem to compare favorably with prospective data from phase II trials (25, 31, 32) addressing the role of concurrent chemotherapy either in combination with modern radiotherapy techniques, or in the setting of dose escalation for various hypofractionation schemes in locally advanced inoperable NSCLC (33–36) (Table 2).This study does not lead to any definitive conclusion on the correlation between overall survival and dose level, but a strong relationship would be expected given that higher dose is known to improve local control. Notwithstanding its retrospective nature and a potential bias due to the accrual of selected (responders-only) patients to the induction chemotherapy, our findings show that high biologically effective dose delivered in a standard time frame may be safely administered with or without chemotherapy, provided that highly conformal radiotherapy techniques are used. More robust clinical trials are needed to confirm this strategy.

**Table 2:** **Comparison of reported series of dose escalation or dose escalated hypofractionated radiotherapy in inoperable non-small cell lung cancer**.

Author	Patients with stage III NSCLC	RT dose	Fraction	Toxicity	Outcomes
Kong (33)	60	63–103 Gy	2.15 Gy	–	5-Years OS, 13%
					Median survival, 19 months
Bradley (34)	83	71–90 Gy	2.10 Gy	No Group 3–4 acute esophagitis	3-Years OS, 26% (IIIA)
				Acute Group ≥3 pneumonitis 6%	15% (IIIB)
				Late Group ≥3 esophagitis 0–8%	
				Late Group ≥3 pneumonitis 0–16%	
Belderbos (35)	42	60–94 Gy	2.25 Gy	No Group 3–4 acute esophagitis	2-Years OS, 24–40% (GTV </≥75 cm^3^)
				Acute Group ≥3 pneumonitis 35%	Median survival, 17 months
				Late Group ≥3 pneumonitis 57%	
				1 Case of late Group 5 esophagitis	
Bral (36)	40	70.5 Gy	2.35 Gy	Acute Group 5 pneumonitis 5%	1-Year OS, 65%
				Acute Group 3 esophagitis 2.5%	2-Years OS, 27%
				Late Group 3 pneumonitis 16%	Median survival, 17 months
				No Group 3–4 late esophagitis	
Adkison (24)	36	57–80.5 Gy	2.28–3.22 Gy	No Group 3–4 acute toxicities	2-Years OS, 46.8%
				Acute Group 2 esophagitis 13%	Median survival, 18 months
				Acute Group 2 pneumonitis 13%	
Current	61	67.5–68.4 Gy	2.25–2.28 Gy	Acute Group 3 esophagitis 3%	1-Year OS, 77%
				Acute Group 3 pneumonitis 12%	2-Years OS, 53%
				Late Group 3 pneumonitis 7%	Median survival:18.6 months (seq Group)
				No Group 3–4 late esophagitis	24.1 Months (conc group)

## Conflict of Interest Statement

The authors declare that the research was conducted in the absence of any commercial or financial relationships that could be construed as a potential conflict of interest.
